# Population structure of *Stemphylium lycopersici* associated with leaf spot of tomato in a single field

**DOI:** 10.1186/s40064-016-3324-9

**Published:** 2016-09-22

**Authors:** Karima Al-Amri, Abdullah M. Al-Sadi, Adel Al-Shihi, Abbas Nasehi, Issa Al-Mahmooli, Mike L. Deadman

**Affiliations:** Department of Crop Sciences, College of Agricultural and Marine Sciences, Sultan Qaboos University, PO Box 34, 123, Al Khod, Muscat, Oman

**Keywords:** *gpd*, ITS, Phylogeny, Population structure, *Solanum lycopersicum*

## Abstract

*Stemphylium lycopersici* is an important pathogen causing leaf spot of tomatoes worldwide. Although much information is available about the pathogen, little is known about dynamics of *S. lycopersici* in tomato fields. Seventy-nine symptomatic leaf samples were collected from two tomato cultivars grown in a farm (Miral and Inbred line). Fungal species associated with the disease were isolated on potato dextrose agar. Seventy-nine isolates were obtained and identified as *S. lycopersici* based on sequence analysis of combined dataset of the internal transcribed spacer and glyceraldehyde-3-phosphate dehydrogenase regions. The 79 isolates were subjected to amplified fragment length polymorphism analysis using three primer combinations. The *Stemphylium lycopersici* population from the two cultivars was found to have a very low level of genetic diversity (H = 0.0948). Cluster analysis showed intermixing of isolates from the two cultivars. In addition, analysis of molecular variance showed the presence of a very low level of genetic differentiation between populations obtained from the two cultivars (F_st_ = 0.0206). These findings indicate the presence of a high rate of gene flow between the two populations and may suggest that the two populations originated from the same inoculum source. The implications of these findings on the management of *Stemphylium*-induced leaf spot of tomatoes are discussed.

## Background

Tomato is among the top vegetable crops in production in different parts of the world (FAO 2015). In Oman, tomato is the top vegetable crop in terms of production (47,812 tones) and area of cultivation (1358 ha). Most (87 %) tomato production is in Al Batihan region, to the north west of the capital area, Muscat. More than 90 % of tomatoes are grown in open fields, while some are planted under greenhouses or shadehouses. Tomato seeds are usually imported from abroad and many of the cultivars are renamed before distribution to farmers. Tomatoes are consumed locally and exported to markets in neighboring countries. Despite the high reliance on this crop, tomato in Oman and in other countries is affected by a number of fungal diseases, the most serious of which are leaf spot and blight diseases.

The genus *Stemphylium* is an important and destructive pathogen causing leaf spot diseases in several agricultural crops (Ellis 1971). In some cases, the disease incidence of tomato leaf spot caused by *Stemphylium* has been reported to reach as high as 100 % (Cedeño and Carrero 1997). *Stemphylium lycopersici* was first described from tomato (Enjoji 1931), and since then, it has been reported in more than 30 host genera worldwide (Ellis and Gibson 1975; Farr and Rossman 2015). *S. solani* is also another species of *Stemphylium* causing grey leaf spot of tomato (Cedeño and Carrero 1997). Both species reproduce asexually through the production of conidia and there is no known teleomorphic stage for both species (Ellis and Gibson 1975; Inderbitzin et al. 2009). In Oman, *S. lycopersici* and *S. solani* have been reported on tomato plants in the Batinah governorate (Waller and Bridge 1978). Symptoms appear as small spots with a yellow halo which eventually become necrotic. It can lead to defoliation and may attack floral parts.

The identification of *Stemphylium* species has relied on sequence analysis of various DNA regions (Câmara et al. 2002; Inderbitzin et al. 2009; Nasehi et al. 2014a, 2015). Previous studies have confirmed that combining the internal transcribed spacers (ITS) and glyceraldehyde-3-phosphate dehydrogenase (*gpd*) regions could help resolve *Stemphylium* to the species level (Câmara et al. 2002; Nasehi et al. 2014a, 2015).

Knowledge of the genetic structure of pathogen populations is essential to predict disease epidemics and to develop effective strategies for disease management (McDonald and Linde 2002a). The genetic variation between isolates or individuals can be assessed using different molecular methods, including amplified fragment length polymorphism (AFLP) fingerprinting (Vos et al. 1995; Al-Sadi 2013; Al-Sadi et al. 2013, 2015).

Population genetic analysis of *S. lycopersici* has focused on the genetic relatedness of isolates from different hosts and geographical locations (Nasehi et al. 2014a). In their study, the polymorphism within *S. lycopersici* isolates was found to be 55 and 69 % using RAPD (random amplified polymorphic DNA) and ISSR (inter simple sequence repeat) markers, respectively. However, no information is available concerning the level of genetic diversity of *S. lycopersici* in a single field or from other locations of the world. This makes our knowledge limited about the level of diversity as well as dynamics and the level of gene flow of the pathogen within the same field.

The main purpose of this study was to analyze the population genetic structure of *S. lycopersici* in a single field. Specific objectives were: (1) to identify *Stemphylium* species associated with leaf spot of tomato in a field in Oman using sequence analysis of combined dataset of the ITS and *gpd* regions, and (2) estimate the levels of genetic diversity and genetic differentiation in *S. lycopersici* populations obtained from two tomato cultivars. Knowledge into these areas will help characterize the potential level of *S. lycopersici* spread within tomato fields and provide a basis for establishing effective management strategies.

## Methods

### Sample collection and isolation

In January 2014, a total of 79 leaf samples developing leaf spot symptoms were collected from two tomato cultivars: Miral and an Inbred line. The cultivars were grown in a completely randomized block design in farm that is located in Barka city, approx. 50 km to the north of capital area of Oman. The number of plants grown in the field was 600 and 420 for Miral and Inbred line, respectively. No pesticides were applied on the two cultivars. The spots were characterized by grey to dark brown and black color, surrounded with yellow hallo. Miral cultivar had a high incidence of the disease, affecting 87 % of the tomato plants grown in the field. However, the Inbred line had a lower incidence level, with 31 % of the plants developing leaf spot symptoms. Out of the 79 collected samples, 55 were from the severely affected cultivar “Miral”, while 24 were from the “Inbred line” which was less affected.

Isolations were established by cutting the symptomatic leaves into small pieces (approx. 5–7 mm). The leaf pieces were disinfected using 1 % sodium hypochlorite for 2–3 min. After that, the pieces were rinsed with sterile distilled water and then blotted dry on sterile filter paper. Isolation of fungi was established on 2.5 % potato dextrose agar (PDA) amended with 10 mg L^−1^ rifampicin and 200 mg L^−1^ ampicillin. The plates were incubated at 25 °C with 12 h photoperiod for 10 days. Colonies of *Stemphylium* were identified to the genus level based on the morphology of spores (Ellis and Gibson 1975) and they were transferred to fresh PDA plates, and pure cultures were established using mycelium tip culture. A total of 79 *Stemphylium* isolates were obtained (one per leaf sample) of which 55 from Miral and 24 from the inbred line.

### DNA extraction

Genomic DNA of *Stemphylium* isolates was extracted using a modified protocol of Lee and Taylor (1990) as described by Al-Sadi et al. (2011). The steps included lysis of fungal cells using a lysis buffer, followed by two steps of phenol–chloroform–isoamyl alcohol (25:24:1), precipitation using sodium acetate and isopropanol and cleaning using 70 % ethanol. The extracted DNA was re-suspended in 100 ml TE buffer (10 mM Tris HCl, 1 mM EDTA) and stored at −20 °C for further use. A Thermo Scientific NanoDrop 2000 was used to check the quality and concentration of the extracted DNA and it was then kept at −20 °C until used.

### Polymerase chain reaction and sequence analysis

The ITS and *gpd* regions of the 79 isolates were amplified using universal primers ITS1 and ITS4 (White et al. 1990) and gpd1 and gpd2 (Berbee et al. 1999), respectively. The PCR reaction mixtures and amplification conditions were as described for the ITS (Al-Sadi et al. 2012b) and gpd2 regions (Nasehi et al. 2014a) using PuReTaq™ Ready-To-Go ™ PCR beads (GE Healthcare, UK). PCR products were resolved on 1.5 % agarose gel under 0.5× Tris–borate–EDTA buffer (TBE) at 120 V for 45 min. The PCR products were sequenced in both directions using the same forward and reverse primers used for PCR amplification. Sequencing was carried out by a commercial sequencing service provider (Macrogen Inc., Seoul, Korea).

To analyze the relationships of the isolates to known *Stemphylium* species, the 79 consensus sequences from this study and 23 sequences of *Stemphylium* species deposited in GenBank by Câmara et al. (2002), Inderbitzin et al. (2009), Kurose et al. (2014) and Hong et al. (2012) were initially aligned using the ClustalW multiple alignment (Thompson et al. 1994). The sequences were checked visually and improved manually where necessary. *Alternaria alternata* (EGS 34-016) was used as an out-group. A neighbor-joining tree was constructed based on the matrix of pairwise distances obtained using the Kimura 2-parameter evolutionary model (Mega 6.0; Tamura et al. 2013). The bootstrap values illustrated on the phylogenetic trees were generated with 1000 replicate heuristic searches. All gaps were treated as missing data.

### AFLP fingerprinting

AFLP was used to examine the genetic diversity within 79 *S. lycopersici* isolates following a modified protocol as described by Al-Sadi et al. (2012a) using FAM-6-labelled *Eco*RI-AGX selective primers. The primer combinations *Eco*RI-AGA/*Mse*I-CAT, *Eco*RIAGT/*Mse*I-CAT, and *Eco*RI-AGT/*Mse*I-CAA were used in this study. DNA restriction and ligation were performed as described by Al-Sadi et al. (2012c). The pre-selective amplification mix consisted of PuReTaq™ Ready-To-Go ™ PCR beads, 0.65 µl *EcoR*I+A (5′-GACTGCGTACCAATTCA-3′), 0.65 µl *Mse*I+C (5′-GATGAGTCCTGAGTAAC-3′), 3.70 µl R/L mix and 20 µl Milli-Q water. The cycling profile was as described by Al-Sadi et al. (2012c). The pre-selective amplification product was diluted by adding 210 μl of TE_0.1_ to the remaining amount. The selective amplification was carried out using the three primer combinations (Table 1). The PRC reaction mixtures and conditions were as described by Al-Sadi et al. (2012c) using PuReTaq™ Ready-To-Go™ PCR beads. The AFLP experiment was carried out twice for each isolate starting from the DNA extraction step.

**Table 1:** Evaluation of the three primer pair combinations utilized to identify and assess genetic diversity within 79 *Stemphylium lycopersici* isolates using AFLP fingerprinting analysis

No.	*Eco*RI	*Mse*I	No. of alleles	No. of Polymorphic alleles	% polymorphism	H^a^
1	AGA	CAA	11	10	91	0.0911
2	AGA	CAT	40	39	98	0.0800
3	AGA	CTG	44	44	100	0.0615

^a^H = Nei’s (1973) gene diversity

### Analysis of AFLP data

The scoring of AFLP data was done as 1 for the presence and 0 for the absence of each locus within the size range of 50–500 base pairs (bp). Genotypic diversity within each population was determined as described by Stoddart and Taylor (1988) followed by scaling it by the number of genotypes (g) (Grünwald et al. 2003). Nei’s gene diversity (Nei 1973), the number of polymorphic loci and genetic distance based on Nei’s (1978) unbiased measures of genetic distance were determined using POPGENE (v 1.32) (Yeh and Boyle 1997). UPGMA (unweighed pair group method with arithmetic mean) analysis used Nei’s unbiased measures of genetic distance for the construction of a dendrogram (NTSYSpc v 2.21m) (Rohlf 2009). Arlequin program version 3.1 (Excoffier et al. 2005) was used for analysis of molecular variance (AMOVA) among the populations obtained from the two tomato cultivars.

## Results

### Identification of *Stemphylium* isolates

**Fig. 1:**
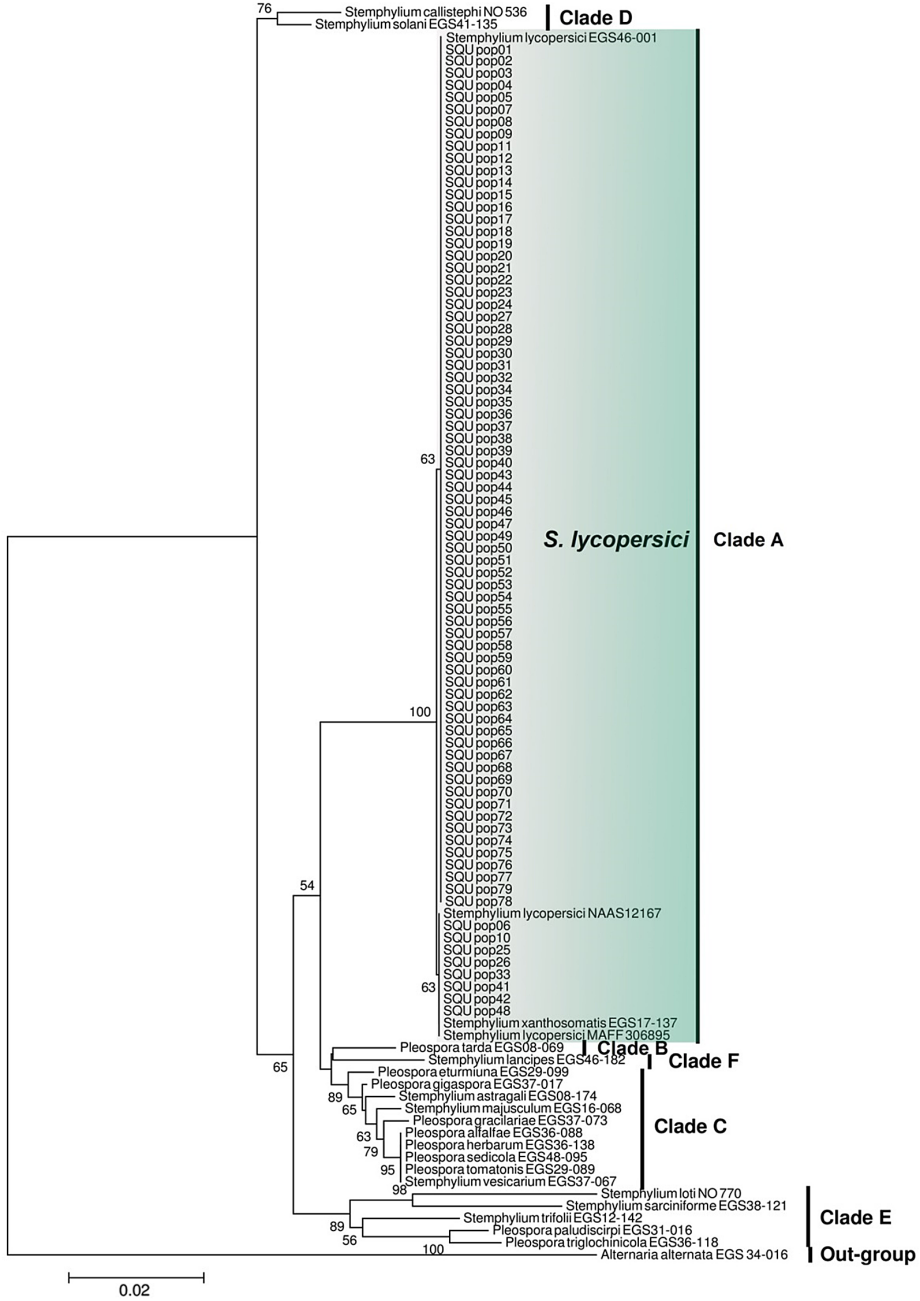
A neighbor-joining tree derived from a ClustalW sequence alignment of combined dataset of the ITS and *gpd* regions of 79 isolates used in this study (SQU pop01–SQU pop79) and other reference *Stemphylium* species. The distances were determined according to Kimura’s two-parameter model. Bootstrap values (>50 %, 1000 replicates) are positioned alongside the branches. *Alternaria alternata* was used as an out-group

PCR amplification of the ITS and *gpd* regions for the 79 isolates (55 form Miral and 24 from an inbred line) produced fragments of ca. 484 and 504 bp, respectively. The final sequence alignment of the combined data sets comprising 103 taxa had 1038 characters, of which 785 characters were constant, 223 were variable, and 94 characters were parsimony informative. The 79 isolates clustered with reference isolates of *S. lycopersici* with a high bootstrap support (Fig. 1), indicating that all the isolates obtained in this study are *S. lycopersici*. The ITS and gpd sequences were deposited in GenBank for two *S. lycopersici* isolates representing the two clades. The GenBank accession numbers are KU556786 (ITS) and KU556787 (gpd) for isolate SQU pop01 and KU556788 (ITS) and KU556789 (gpd) for isolate SQU pop06.

### Genetic and genotypic analysis

The three primer combinations E+AGA/M+CAA, E+AGA/M+CAT and E+AGA/M+CTG generated 11, 40 and 44 alleles, of which 10, 39 and 44 alleles were polymorphic, respectively. Nei’s gene diversity (1973) was found to be low for the three primer combinations (Table 1).

Population genetic analysis of the population from ‘Miral’ cultivar and the ‘inbred line’ cultivar showed that both populations have a very low level of genetic diversity (0.0708 and 0.0677, respectively). The populations from ‘Miral’ cultivar the ‘inbred line’ cultivar produced 55 and 24 genotypes, respectively. The total number of polymorphic alleles was 93 within all isolates (Table 2).

**Table 2:** Genetic analysis of 79 *Stemphyllium lycopersici* isolates obtained from the two tomato cultivars Miral and Inbred line using AFLP fingerprinting analysis

Cultivar	N	NPL	PPL	*G*	% Ĝ/g	H
Miral	55	75	81	55	100	0.0708
Inbred line	24	60	65	24	100	0.0677
All	79	93	98	79	100	0.0948

N is the sample size, NPL is the number of polymorphic loci, PPL is the percentage of polymorphic loci (out of 98), *g* is the number of different genotypes recovered, % Ĝ/*g* is the percentage of maximum diversity obtained in each population, and H is Nei (1973) gene diversity

### Cluster and AMOVA analyses

**Fig. 2:**
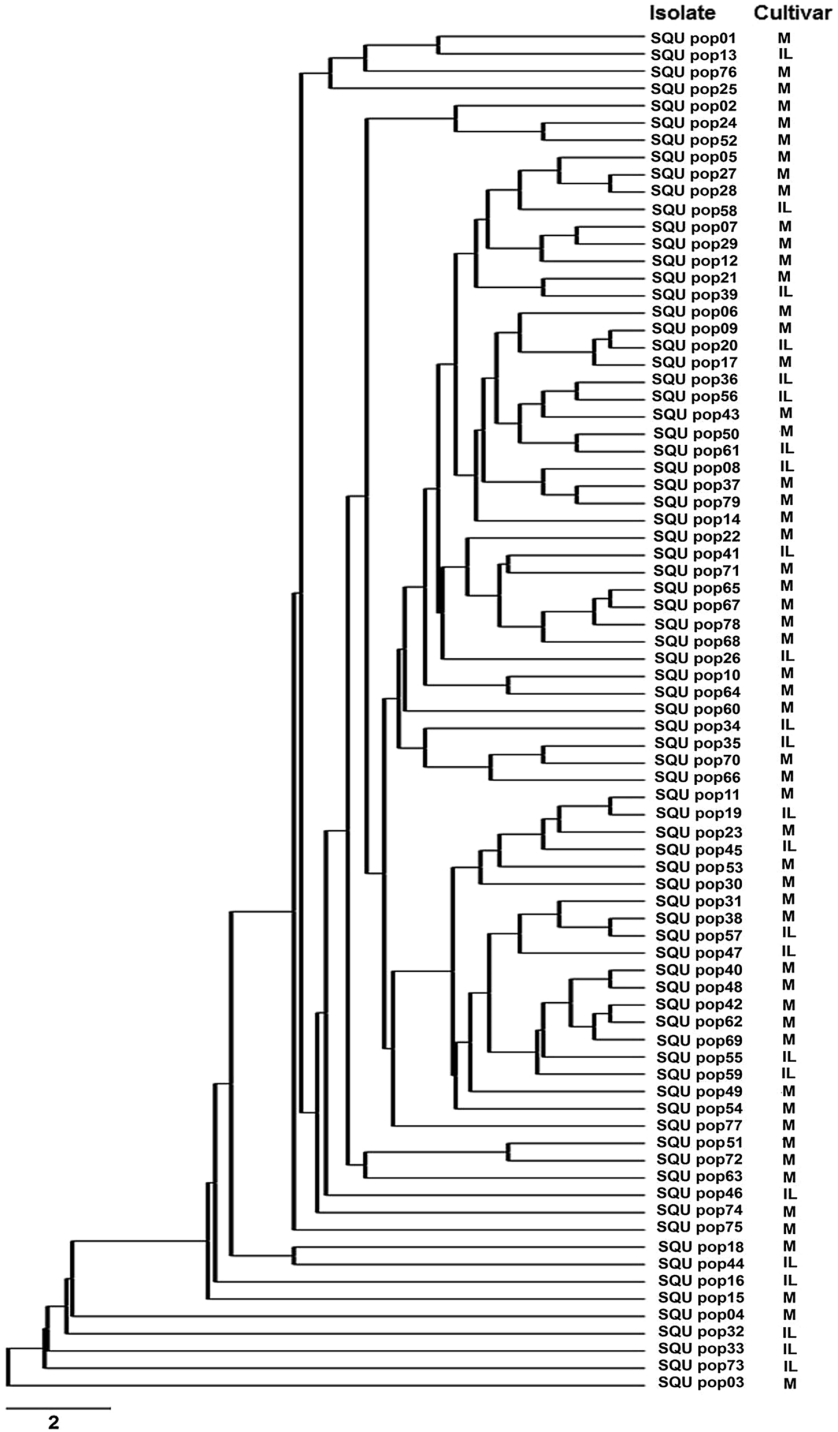
UPGMA dendrogram illustrating Nei’s (1978) genetic distance of *Stemphyllium lycopersici* isolates (SQU pop01–SQU pop79) obtained from the two tomato cultivars Miral (M) and Inbred line (IL) based on AFLP fingerprinting analysis

Pairwise analysis of genetic differentiation indicated the presence of a very low levels of genetic differentiation between the two populations (F_st_ = 0.0206). The rate of gene flow of isolates between the two cultivars was high (N_m_ = 23.8). These data were consistent with UPGMA analysis, which grouped 79 *S. lycopersici* isolates obtained from the two tomato cultivars into subclusters of 79 AFLP genotypes (Fig. 2). Cluster analysis revealed that isolates obtained from the two tomato cultivars were intermixed together, and no relationship was observed between the clustering of the isolates and the cultivars from which they were obtained.

## Discussion

Isolations from tomato leaves revealed the association of *Stemphylium lycopersici* with the leaf spot symptoms. This fungal species has been reported as a causal agent of leaf spot on tomato in different countries (Enjoji 1931; Ellis and Gibson 1975; Min et al. 1995; Nasehi et al. 2014b). Phylogenetic analysis of the 79 isolates with reference isolates revealed that the same five clades, A–E, were delineated as indicated by Câmara et al. (2002), and Clade F was also congruent with that of Inderbitzin et al. (2009) (Fig. 1). The analysis showed that all 79 isolates clustered in clade A which included reference species of *S. lycopersici* (EGS 46-001) and *S. xanthosomatis* (EGS 17-137) by a strong bootstrap value (100 %). However, Câmara et al. (2002) suggested that *S. xanthosomatis* is a synonym of *S. lycopersici*. Our data therefore show that all the isolates which were obtained in this study are *S. lycopersici.*

Tomatoes have been grown in the field for at least the last 10 years. Leaf spot symptoms have been observed in the field in the past, but at low levels. However, the incidence was very high during this season. Although this could be related to growing the susceptible cultivar “Miral” for the first time in the farm, this hypothesis needs to be investigated in future studies by doing pathogenicity tests on this cultivar and the other cultivars grown in the farm.

A low level of genetic diversity was observed within populations of *S. lycopersici* from the two tomato cultivars. This could be related to several factors. Firstly, the population of *S. lycopersici* in the farm could have originated from a single source in the past. Fungal species originating from a single source have been reported to have a relatively low level of genetic diversity (Al-Sa’di et al. 2008; Al-Sadi et al. 2013). This fungus is mainly transmitted via wind for short distances (Rossi et al. 2005) and it is unlikely to be transmitted frequently from other fields due to the separation of this field from other fields by more than 1 km. The second factor resulting in the low genetic diversity could be the low incidence of the disease in the field in the past, which could have kept the rates of reproduction and evolution of *S. lycopersici* at low levels (McDermott and McDonald 1993; McDonald and Linde 2002b). However, the contribution of resistance of the previously cultivated tomato cultivars in lowering the reproduction rate of *S. lycopersici* in the field is a hypothesis which deserves investigation in future studies.

No relationship was found between clustering of *S. lycopersici* isolates and the cultivars from which they were obtained. These findings are in agreement with the results of Mehta (2001), who did not observe any clear relationship between genetic variability of *S. solani* isolates and cotton and tomato cultivars from which the isolates were collected. They are also in agreement with findings which showed the lack of relationship between clustering of *Ceratocystis radicicola* isolates and the cultivars from which they were obtained (Al-Sadi 2013).

The low level of genetic differentiation and the presence of high gene flow among the two *S. lycopersici* populations from tomato cultivars is consistent with the nature of this pathogen which is airborne and can be transmitted by several means including wind and rain splash (Rossi et al. 2005; Ahmad et al. 2016). The high rate of movement of *S. lycopersici* within the tomato field represents a challenge for the management of the pathogen. Management strategies aimed at eliminating the spread of the pathogen may not be very effective during windy or rainy seasons. However, continuous inspection of primary inoculum infestations in fields and removal of primary inoculum may help reduce the chance of disease establishment. The use of resistant cultivars is an important strategy. Resistance genes have been incorporated in tomatoes against several pathogens, including *S. lycopersici* (Parlevliet 2002). This could be one of the reasons why the severity of grey leaf spot of tomato has been low in the field over the last 10 years. However, chemical control may be necessary in some cases to prevent disease levels from reaching economic thresholds (de Miranda et al. 2010).

## Conclusion

The combined dataset of the ITS and *gpd* regions helped identify 79 *S. lycopersici* isolates to the species level. Population genetic analysis indicated frequent movement of *S. lycopersici* within fields. This may result in high disease epidemics under the most favorable environmental conditions. Since temperature, wind speed, tomato cultivars and other factors may affect the population structure of fungi (McDermott and McDonald 1993; McDonald and McDermott 1993), future studies should address the population structure of *S. lycopersici* under different growing conditions. In addition, studies are required to address pathogen specialization on different cultivars.

## References

[CR1] Ahmad A, Thomas GJ, Barker SJ, Macleod WJ (2016). Genotype resistance, inoculum source and environment directly influence development of grey leaf spot (caused by *Stemphylium* spp.) and yield loss in narrow-leafed lupin (*Lupinus angustifolius*). Crop Pasture Sci.

[CR2] Al-Sadi AM (2013). Phylogenetic and population genetic analysis of *Ceratocystis radicicola* infecting date palms. J Plant Pathol.

[CR3] Al-Sadi AM, Al-Said FA, Al-Kiyumi KS, Al-Mahrouqi RS, Al-Mahmooli IH, Deadman ML (2011). Etiology and characterization of cucumber vine decline in Oman. Crop Prot.

[CR4] Al-Sadi A, Al-Ghaithi A, Al-Balushi Z, Al-Jabri A (2012). Analysis of diversity in Pythium aphanidermatum populations from a single greenhouse reveals phenotypic and genotypic changes over 2006 to 2011. Plant Dis.

[CR5] Al-Sadi AM, Al-Jabri AH, Al-Mazroui SS, Al-Mahmooli IH (2012). Characterization and pathogenicity of fungi and oomycetes associated with root diseases of date palms in Oman. Crop Prot.

[CR6] Al-Sadi AM, Al-Moqbali H, Al-Yahyai R, Al-Said F, Al-Mahmooli I (2012). AFLP data suggest a potential role for the low genetic diversity of acid lime (*Citrus aurantifolia*) in Oman in the outbreak of witches’ broom disease of lime. Euphytica.

[CR7] Al-Sadi AM, Al-Wehaibi AN, Al-Shariqi RM, Al-Hammadi MS, Al-Hosni IA, Al-Mahmooli IH, Al-Ghaithi AG (2013). Population genetic analysis reveals diversity in *Lasiodiplodia* species infecting date palm, Citrus, and mango in Oman and the UAE. Plant Dis.

[CR8] Al-Sadi AM, Al-Masoodi RS, Al-Ismaili M, Al-Mahmooli IH (2015). Population structure and development of resistance to hymexazol among *Fusarium solani* populations from date palm, Citrus and cucumber. J Phytopathol.

[CR9] Al-Sa’di AM, Drenth A, Deadman ML, de Cock AWAM, Al-Said FA, Aitken EAB (2008). Genetic diversity, aggressiveness and metalaxyl sensitivity of *Pythium spinosum* infecting cucumber in Oman. J Phytopathol.

[CR10] Berbee ML, Pirseyedi M, Hubbard S (1999). Cochliobolus phylogenetics and the origin of known, highly virulent pathogens, inferred from ITS and glyceraldehyde-3-phosphate dehydrogenase gene sequences. Mycologia.

[CR11] Câmara MP, O’Neill NR, Van Berkum P (2002). Phylogeny of *Stemphylium* spp. based on ITS and glyceraldehyde-3-phosphate dehydrogenase gene sequences. Mycologia.

[CR12] Cedeño L, Carrero C (1997). First report of tomato gray leaf spot caused by *Stemphylium solani* in the Andes region of Venezuela. Plant Dis.

[CR13] de Miranda BEC, Boiteux LS, Reis A (2010). Identification of Solanum (section Lycopersicon) accessions with resistance to *Stemphylium solani* and *S. lycopersici*. Hortic Bras.

[CR14] Ellis MB (1971). Dematiaceous hyphomycetes.

[CR15] Ellis M, Gibson IAS (1975) *Stemphylium lycopersici*. IMI Descriptions of Fungi and Bacteria Set 48: sheet 471

[CR16] Enjoji S (1931). Two diseases of tomato (2). J Plant Prot.

[CR17] Excoffier L, Laval G, Schneider S (2005). Arlequin (version 3.0): an integrated software package for population genetics data analysis. Evol Bioinform Online.

[CR18] FAOSTAT (2015) FAO. http://faostat3.fao.org/browse/rankings/countries_by_commodity/E. Accessed 2015

[CR19] Farr D, Rossman A (2015) Fungal databases, systematic mycology and microbiology laboratory, ARS, USDA. Retrieved December 12, from http://nt.ars-grin.gov/fungaldatabases/

[CR20] Grünwald NJ, Goodwin SB, Milgroom MG, Fry WE (2003). Analysis of genotypic diversity data for populations of microorganisms. Phytopathology.

[CR21] Hong SK, Choi HW, Lee YK, Shim HS, Lee SY (2012). Leaf spot and stem rot on Wilford Swallowwort caused by *Stemphylium lycopersici* in Korea. Mycobiology.

[CR22] Inderbitzin P, Mehta YR, Berbee ML (2009). Pleospora species with *Stemphylium anamorphs*: a four locus phylogeny resolves new lineages yet does not distinguish among species in the *Pleospora herbarum* clade. Mycologia.

[CR23] Kurose D, Hoang LH, Furuya N, Takeshita M, Sato T, Tsushima S, Tsuchiya K (2014). Pathogenicity of Stemphylium lycopersici isolated from rotted tobacco seeds on seedlings and leaves. J Gen Plant Pathol.

[CR24] Lee SB, Taylor JW, Innis MA, Gelfand DH, Sninsky JJ, White TJ (1990). Isolation of DNA from fungal mycelia and single spores. PCR protocols: a guide to methods and applications.

[CR25] McDermott JM, McDonald BA (1993). Gene flow in plant pathosystems. Annu Rev Phytopathol.

[CR26] McDonald BA, Linde C (2002). Pathogen population genetics, evolutionary potential, and durable resistance. Annu Rev Phytopathol.

[CR27] McDonald BA, Linde C (2002). Pathogen population genetics, evolutionary potential, and durable resistance. Annu Rev Phytopathol.

[CR28] McDonald BA, McDermott JM (1993). Population genetics of plant pathogenic fungi. Bioscience.

[CR29] Mehta Y (2001). Genetic diversity among isolates of Stemphylium solani from cotton. Fitopatol Bras.

[CR30] Min J, Kim B, Cho K, Yu S (1995). Grey leaf spot caused by Stemphylium lycopersici on tomato plants. Korean J Plant Pathol (Korea Republic).

[CR31] Nasehi A, Kadir J-B, Nasr-Esfahani M, Abed-Ashtiani F, Wong M-Y, Rambe S-K, Golkhandan E (2014). Analysis of genetic and virulence variability of *Stemphylium lycopersici* associated with leaf spot of vegetable crops. Eur J Plant Pathol.

[CR32] Nasehi A, Kadir JB, Esfahani MN, Mahmodi F, Golkhandan E, Akter S, Ghadirian H (2014). Cultural and physiological characteristics of *Stemphylium lycopersici* causing leaf blight disease on vegetable crops. Arch Phytopathol Plant Prot.

[CR33] Nasehi A, Kadir J, Nasr-Esfahani M, Abed-Ashtiani F, Golkhandan E, Ashkani S (2015). Identification of the new pathogen (*Stemphylium lycopersici*) causing leaf spot on Pepino (*Solanum muricatum*). J Phytopathol.

[CR34] Nei M (1973). Analysis of gene diversity in subdivided populations. Proc Natl Acad Sci.

[CR35] Nei M (1978). Estimation of average heterozygosity and genetic distance from a small number of individuals. Genetics.

[CR36] Parlevliet JE (2002). Durability of resistance against fungal, bacterial, and viral pathogens; present situation‎. Euphytica.

[CR37] Rohlf FJ (2009). NTSYSpc: numerical taxonomy system. ver. 2.21c. Exeter Software.

[CR38] Rossi V, Bugiani R, Giosué S, Natali P (2005). Patterns of airborne conidia of *Stemphylium vesicarium*, the causal agent of brown spot disease of pears, in relation to weather conditions. Aerobiologia.

[CR39] Stoddart JA, Taylor JF (1988). Genotypic diversity: estimation and prediction in samples. Genetics.

[CR40] Tamura K, Stecher G, Peterson D, Filipski A, Kumar S (2013). MEGA6: molecular evolutionary genetics analysis version 6.0. Mol Biol Evol.

[CR41] Thompson JD, Higgins DG, Gibson TJ (1994). CLUSTAL W: improving the sensitivity of progressive multiple sequence alignment through sequence weighting, position-specific gap penalties and weight matrix choice. Nucleic Acids Res.

[CR42] Vos P, Hogers R, Bleeker M, Reijans M, Van de Lee T, Hornes M, Friters A, Pot J, Paleman J, Kuiper M (1995). AFLP: a new technique for DNA fingerprinting. Nucleic Acids Res.

[CR43] Waller J, Bridge J (1978). Plant diseases and nematodes in the Sultanate of Oman. PANS.

[CR44] White TJ, Bruns T, Lee S, Taylor J (1990). Amplification and direct sequencing of fungal ribosomal RNA genes for phylogenetics. PCR Prot Guide Methods Appl.

[CR45] Yeh F, Boyle T (1997). Population genetic analysis of codominant and dominant markers and quantitative traits. Belg J Bot.

